# HMGB1 Activates Myeloid Dendritic Cells by Up-Regulating mTOR Pathway in Systemic Lupus Erythematosus

**DOI:** 10.3389/fmed.2021.636188

**Published:** 2021-06-07

**Authors:** Xinghui Song, Hui Zhang, Yun Zhao, Yuanzhen Lin, Qiya Tang, Xiu Zhou, Xiaoning Zhong

**Affiliations:** ^1^Department of Respiratory and Critical Care Medicine, The First Affiliated Hospital of Guangxi Medical University, Nanning, China; ^2^Department of Rheumatism and Immunology, The Fourth Affiliated Hospital of Guangxi Medical University, Liuzhou, China

**Keywords:** dendritic cells, HMGB1, signal transduction pathway, systemic lupus erythematosus, mTOR inhibitor

## Abstract

Research has shown that HMGB1 can activate dendritic cells (DCs), but its molecular mechanisms are not clear. In this study, we reported that the myeloid dendritic cells (mDCs) were activated in the peripheral blood of SLE patients, and the activation of mDCs was associated with the up-regulation of HMGB1 and mTOR. After stimulated by HMGB1, expression of mTOR and its substrates P70S6K and 4EBP1 in dendritic cells increased considerably (*P* < 0.01). The expression of HLA-DR, CD40, and CD86 on dendritic cells also significantly increased following these stimuli (*P* < 0.01). In addition, stimulation with HMGB1 enhanced cytokine (IL-1β, IL-6, and TNF-a) production in dendritic cells. In contrast, the HMGB1-mediated expression of HLA-DR, CD40, and CD86 on dendritic cells and production of IL-1β, IL-6, and TNF-α were reduced by rapamycin. Rapamycin can inhibit HMGB1-induced activation of mDCs and secretion of pro-inflammatory cytokines. These findings indicated that HMGB1activates mDCs by up-regulating the mTOR pathway in SLE.

## Introduction

Systemic lupus erythematosus (SLE) is a severe, debilitating autoimmune disease that affects multiple organs and body systems. The prevalence of SLE worldwide is estimated to be as high as 150 per 100,000 individuals (1). The disease is characterized by autoantibodies against nuclear antigens (ANA), which result from immune system deregulation (2). Although research has been done, the pathogenesis of SLE is not yet fully understood.

Dendritic cells (DCs) play an essential role in bridging the innate and adaptive immune systems. DCs are antigen-presenting cells displaying the unique capability to activate naïve T cells. DCs can also respond to encounter pathogens by producing inflammatory mediators, including proinflammatory cytokines (3). Because of these complex roles, an imbalance in DC functions can cause a defective or exaggerated immune response and tissue damage. Research has shown that HMGB1, a non-histone nuclear protein, can induce immune responses and inflammatory responses that are relevant for the pathogenesis of SLE (4). For example, recent evidence indicates that HMGB1 is responsible for producing proinflammatory cytokines, which is a well-established damage associated molecular pattern (DAMP) (5–7). HMGB1 is likely to be released from activated immune cells such as dendritic cells (DCs) in inflammation or injury (8). When released, HMGB1 participates in the secretion of downstream proinflammatory cytokines *via* binding to cell surface receptors such as receptor of advanced glycation end products (RAGE), TLR2, and TLR4, contributing to the occurrence and development of diverse inflammatory diseases and autoimmune diseases (9–14).

One signaling pathway that has been the subject of current research in SLE is the mechanistic target of rapamycin (mTOR). Recent studies have found that activation of mTOR in both the immune system (15, 16) and non-traditional parenchymal organs (e.g., the liver) precedes the onset of disease and represents early manifestations of pathogenesis (17). A common finding in T cells, B cells, macrophages, hepatocytes, and renovascular cells is mTOR activation (18). But little research has been done on mTOR activation in dendritic cells in SLE. Therefore, this study aimed to assess the potential role of mTOR in the activation of myeloid dendritic cells (mDCs) triggered by HMGB1 in patients with SLE.

## Materials and Methods

### Design

The study used a case-control design to examine whether HMGB1 can contribute to the up-regulation of the mTOR pathway of dendritic cells in patients with SLE. We collected peripheral blood samples from patients with SLE and healthy controls (HCs). We quantified the level of HMGB1 with ELISA and analyzed mDCs from peripheral blood with flow cytometry. Next, we stimulated mDCs from SLE with HMGB1. The expression of mTOR and its substrates, HLA-DR, costimulatory molecules of dendritic cells, and cytokine synthesis were measured. These indexes were measured again after blocking the mTOR pathway with rapamycin.

### Participants

We recruited 35 patients with SLE, who fulfilled the American College of Rheumatology (ACR) revised criteria for the classification of SLE (19), and 20 healthy controls. SLE disease activity index 2000 (SLEDAI-2K) (20) was determined in the blood sampling. Patients with SLE were recruited from the Department of Rheumatism and Immunology, the First Affiliated Hospital of Guangxi Medical University. All patients were newly diagnosed and did not receive any corticosteroids or immunosuppressive treatment at the time of the blood collection. Healthy adults matched for age and sex were enrolled from blood donors. The study was approved by the Medical Ethical Committee of the First Affiliated Hospital of Guangxi Medical University (NO.2017-KY-国基-111) and the Medical Ethical Committee of the Fourth Affiliated Hospital of Guangxi Medical University (NO. KY2018083), China. All subjects signed the informed consent.

### Quantitation of HMGB1

HMGB1 in serum samples of 35 SLE patients and 20 healthy controls were analyzed with ELISA kits (CUSABIO) according to the manufactural protocol. Plates were read at an absorbance of 450 nm (A450) using a Sunrise microplate reader. All measurements were carried out in duplicate.

### Analysis mDCs From Peripheral Blood of SLE With Flow Cytometry

Peripheral blood mononuclear cells (PBMC) were isolated from the peripheral blood samples of SLE patients by using Lymphoprep™ (STEMCELL). Freshly isolated PBMCs were stained for surface marker FITC anti-human Lineage Cocktail (CD3, CD14, CD19, CD20, CD56) (BD Pharmingen), PerCp anti-human HLA-DR (BD Pharmingen), PE anti-human CD11c (BD Pharmingen), APC anti-human CD40 (BD Pharmingen) or APC anti-human CD86 (BD Pharmingen). After surface staining, cells were fixed/permeabilized with fixation/permeabilization solution (Cytofix/Cytoperm™; BD Pharmingen), and stained with p-mTOR-Alexa 647 (BD Pharmingen) for 30 min at 4°C. Cells were then washed with 1 × Perm/Wash Buffer (BD Pharmingen) and resuspended in PBS + 2% FBS for flow cytometric analysis. Flow cytometry was performed on a BD FACS Canto II (BD Biosciences) and data were analyzed using FCS Express 4 software (De Novo Software, Los Angeles, CA).

### Preparation and Stimulation of mDCs

Whole blood samples from 20 patients with SLE were collected using ACD tubes (BD Vacutainer). Peripheral blood mononuclear cells (PBMCs) were isolated using Lymphoprep™ (STEMCELL). Cells were cultured in RPMI-1640 supplemented with 10% FBS at a density of 2 × 10^6^ cells/ml in 6-well tissue culture plates. After PBMC were cultured for 3 h, the liquid in the containers was discarded. Then RPMI-1640 was added for further culture, supplemented with 10% FBS, recombinant human GM-CSF (100 ng/ml; PeproTech), and recombinant human IL-4 (100 ng/ml; PeproTech). On days 2 and 4 of culture, the supernatant was removed and replaced with fresh medium containing hGM-CSF and hIL-4. All cultures were incubated at 37°C in 5% humidified CO_2_. After seven days of culture, more than 95% of the cells expressed CD11c +, the characteristic DC-specific markers, as determined by FACS (Supplementary Figure 1).

To determine the effect of HMGB1 on mDC activation, primary mDCs were stimulated with HMGB1 (1 ug/ml; Peprotech) on Day 6 of culture. Cells were then collected after 24 h.

To block the mTOR pathway, we added different concentrations of rapamycin (10, 20, and 40 ng/ml) to primary mDCs on Day 5 of culture, and then stimulated with HMGB1 as described above.

### Measurement of Cytokine Production and Surface Molecules of mDCs

Levels of various cytokines (IL-1β, IL-6, and TNF-α) in the supernatant of mDCs cultures were quantified using ELISA analysis (BD Biosciences).

The surface molecules on dendritic cells were measured by flow cytometry. Cultured mDCs were stained for surface markers PerCp anti-human HLA-DR, PE anti-human CD11c, APC anti-human CD40, or APC anti-human CD86. All antibodies and isotype control were purchased from BD Pharmingen. Cell surface staining was performed according to the standard procedures.

### The Expression of mTOR in mDCs by RT-PCR

Total cellular RNAs were isolated from DCs using AxyPrep Multisource Total RNA Miniprep Kit (Axygen), and RNA samples were transformed into complementary DNA (cDNA) using RevertAid First-strand cDNA Synthesis kit (Thermo) according to the manufacturer's instructions. Quantitative PCR was performed on selected targets (mTOR, P70S6K, and 4EBP1) using pre-developed primers and probes (Sangon Biotech) on the QuantStudio (TM) real-time PCR software System. β-actin was used as an internal control. Primer sequences were the following: β-ACTIN, 5′-CCT GGC ACC CAG CAC AAT-3′ and 5′ -GGG CCG GAC TCG TCA TAC-3′; mTOR, 5′-ACT GGA GGC TGA TGG ACA CA-3′ and 5′-GGC TCT CCA AGT TCC ACA CC-3′; P70S6K, 5′-CAT CGG CAC CAC TTC CAA TA-3′ and 5′-TTC ATA CGC AGG TGC TCT GG-3′; 4EBP1, 5′-TCG GAA CTC ACC TGT GAC CA-3′ and 5′-GCT CAT CAC TGG AAG GGC TG-3′. Expression of mTOR, P70S6K, and 4EBP1 were calculated as described by the manufacturer.

### Statistical Analysis

Graph Pad Prism version 7 (GraphPad Software Inc., San Diego, CA, USA) was used to perform comparisons between different groups and to generate figures. Calculations were based on a 95% confidence interval (CI). *P*-values < 0.05 were considered significant. All data were expressed as the mean ± SD. Student's *t*-test was used for continuous variables that were normally distributed. The Mann–Whitney *U*-test was used for continuous variables that were not normally distributed.

## Result

We included 35 subjects with SLE and 20 healthy controls. All patients were newly diagnosed with SLE and did not receive any corticosteroids or immunosuppressive treatment at the time of the blood draw. Most patients with SLE were women (29/35, 83%), with a mean age of 40 ± 13.27 years. They had a SLEDAI score of 9.9 ± 4. Common disease activity at the time of inclusion was renal (66%). Healthy controls (HCs) consisted of 20 healthy individuals matched for sex and age. Other characteristics of the patients are summarized in Supplementary Table 1.

### mDCs Were Activated in the Peripheral Blood of SLE Patients, and the mDCs Activation Was Associated With the Up-Regulation of HMGB1 and mTOR

**Figure 1:**
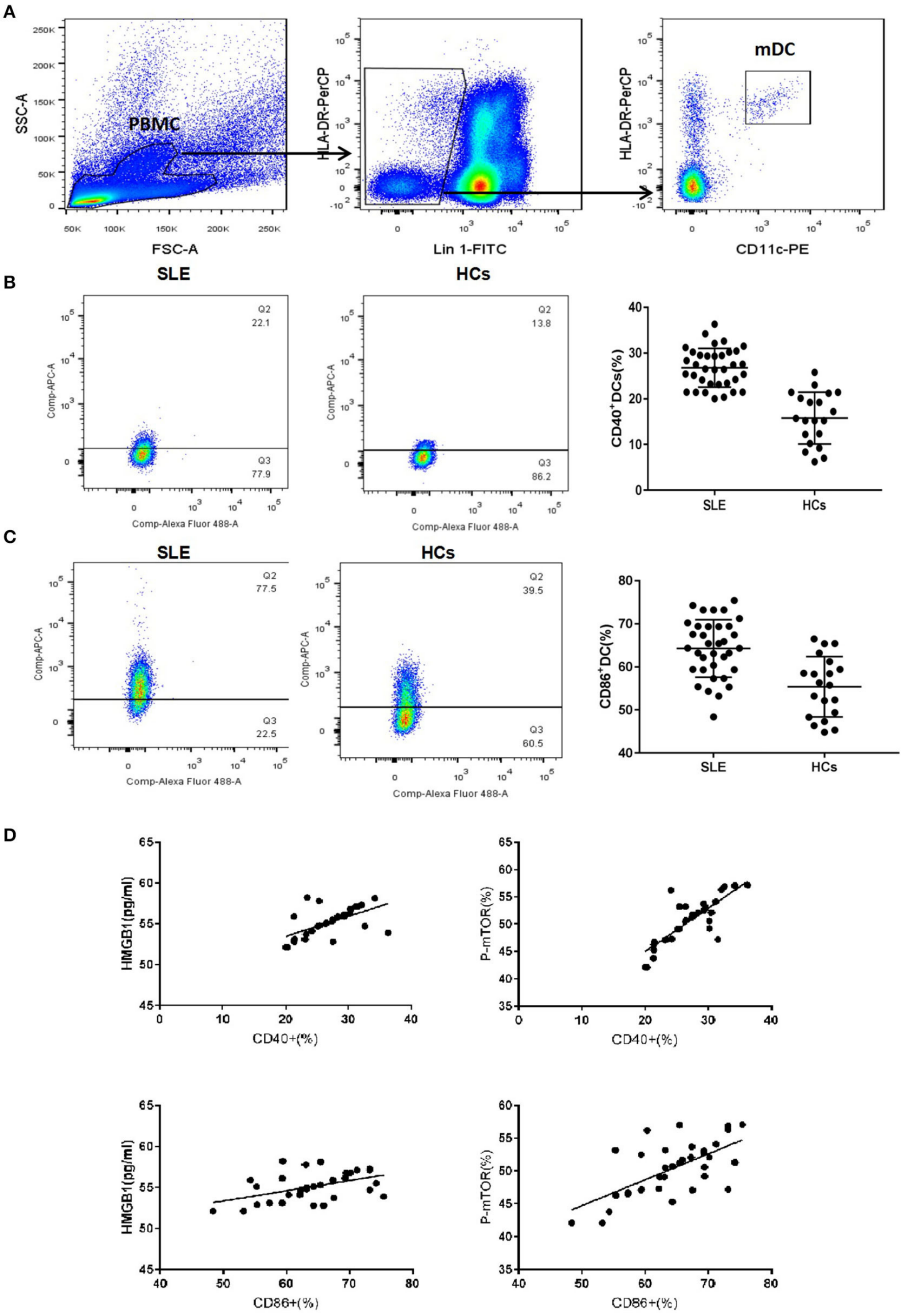
mDCs phenotype of SLE and healthy control group. **(A)** Gating strategy for flow cytometry analysis of mDCs. **(B)** The mDC in peripheral blood of SLE patients to express more CD40 than HCs (26.79 ± 4.23% vs. 15.78 ± 5.68%, *t* = 8.185, *p* = 0.000). **(C)** The mDC in peripheral blood of SLE patients to express more CD86 than HCs (64.27 ± 6.68% vs. 55.38 ± 7.01%, *t* = 4.664, *p* = 0.000). **(D)** The levels of CD40 and CD86 were positively correlated with the levels of HMGB1 (CD40: *r* = 0.600, *P* = 0.001; CD86: *r* = 0.478, *P* = 0.004) and p-mTOR in mDCs (CD40: *r* = 0.812, *P* = 0.001; CD86: *r* = 0.640, *P* = 0.001).

Compared to healthy controls (HCs), mDC in the peripheral blood of SLE patients expressed more CD40 and CD86 (Figures 1A–C). The levels of CD40 and CD86 were positively correlated with levels of HMGB1 and p-mTOR in mDCs (Figure 1D).

**Figure 2:**
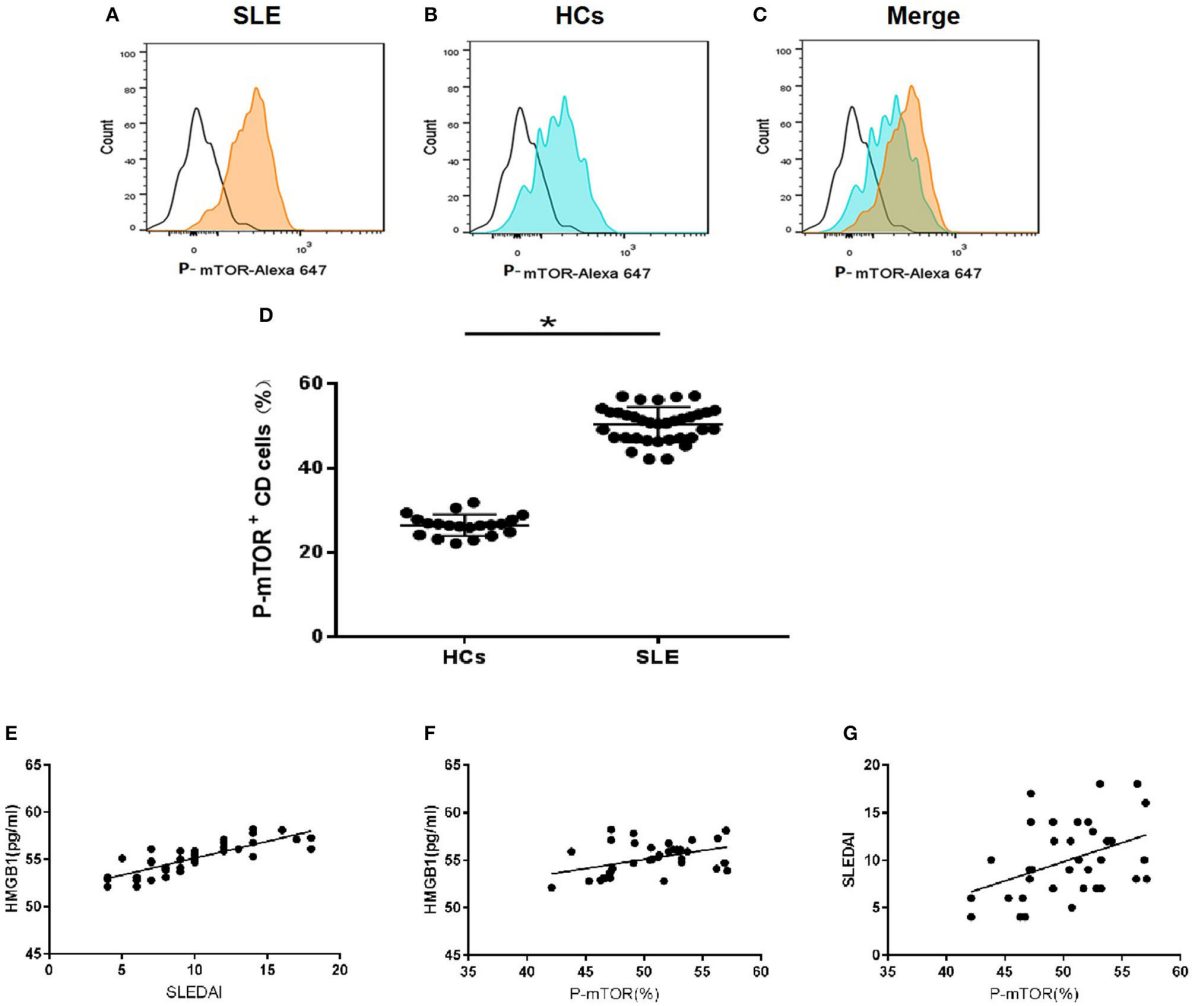
HMGB1 was unregulated in SLE patients and positively correlated with levels of p-mTOR in mDCs. **(A)** The level of p-mTOR in DCs from SLE patients; **(B)** The level of p-mTOR in DCs from healthy controls (HCs); **(C)** The merged figure of A with B; **(D)** The level of p-mTOR in DCs from SLE patients was higher than HCs (50.35 ± 0.696% vs. 26.43 ± 0.565%, *P* = 0.027); *P < 0.05. **(E)** The correlation between serum HMGB1 concentrations and SLE disease activity index score (SLEDAI) in SLE patients. In peripheral blood, HMGB1 levels were positively correlated with SLEDAI scores (*r* = 0.817, *p* < 0.001). **(F)** The correlation between the SLEDAI and p-mTOR in mDCs in peripheral blood of SLE patients. Levels of p-mTOR in mDCs were positively correlated with the SLEDAI (*r* = 0.417, *p* < 0.0128). **(G)** The correlation between the levels of HMGB1 and p-mTOR in mDCs in peripheral blood of SLE patients. Levels of p-mTOR in mDCs positively correlated with the levels of HMGB1 in peripheral blood (*r* = 0.446, *p* < 0.0072).

Mean serum level of HMGB1 in SLE patients was significantly higher than that in healthy controls (HCs) (66.570 ± 17.995 pg/ml vs. 53.265 ± 8.727 pg/ml, *p* < 0.001). HMGB1 levels were positively correlated with SLEDAI scores (*r* = 0.817, *p* < 0.001, Figure 2E).

The expression of p-mTOR in mDCs increased significantly in SLE patients (Figures 2A–D). The levels of p-mTOR in mDCs were positively correlated with SLEDAI scores (*r* = 0.943, *p* < 0.001, Figure 2F) and the levels of HMGB1 in peripheral blood (*r* = 0.805, *p* < 0.001, Figure 2G), respectively.

### HMGB1 Activated Myeloid Dendritic Cells and Up-Regulated mTOR Pathways in mDCs From SLE Patients

**Figure 3:**
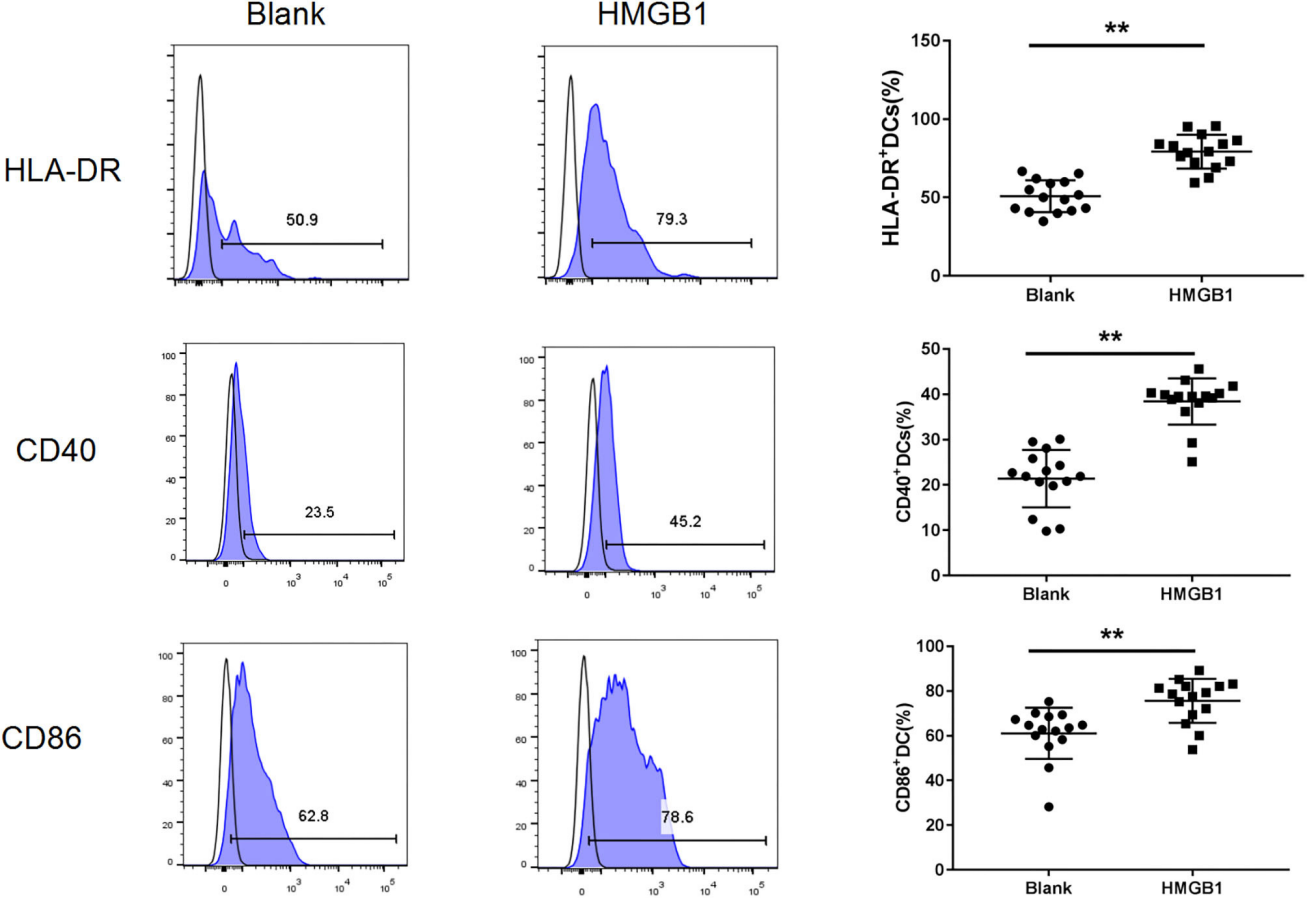
HMGB1 promoted activation of mDCs from SLE patients. Flow cytometry analysis of HLA-DR, CD40, and CD86, surface expression on each group mDCs from SLE patients. The expression of HLA-DR (79.27 ± 10.78% vs. 50.77 ± 10.14%, *t* = 7.455, *P* = 0.00), CD40 (38.42 ± 5.10% vs. 21.41 ± 6.33%, *t* = 8.102, *P* = 0.000) and CD86 (75.63.42 ± 9.80% vs. 61.07 ± 11.48%, *t* = 3.735, *P* = 0.001) were up-regulated on mDCs after stimulation with HMGB1.**P < 0.001.

**Figure 4:**
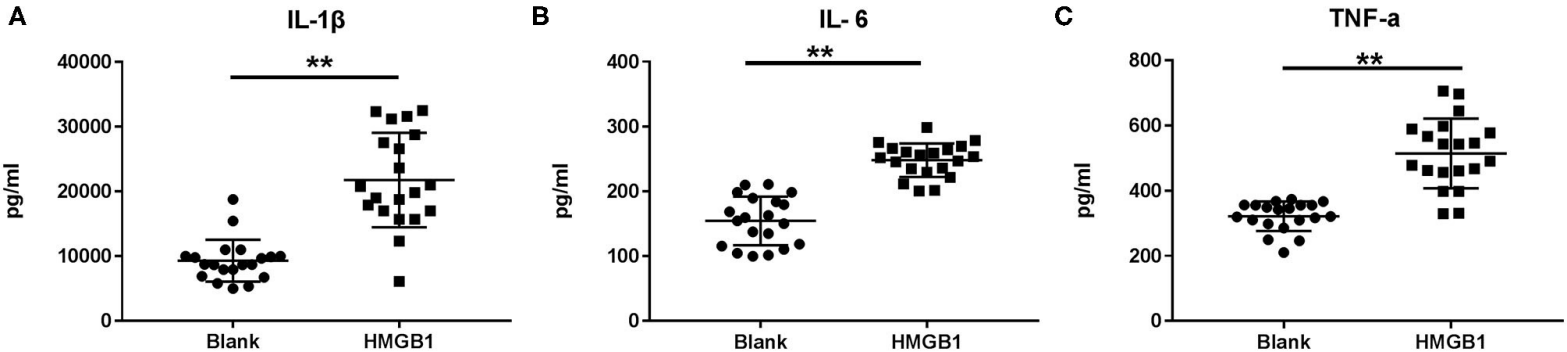
Cytokine production following stimulation of HMGB1 measured by ELISA. When stimulated with HMGB1, the mDCs from systemic lupus erythematosus (SLE) patients produced significantly more IL-1β **(A)**, IL-6 **(B)**, and TNF-α **(C)** than the Blank. Data are presented as the median and interquartile range (IL-1β: 21745 ± 1632.45pg/ml vs. 9282.6 ± 723.68 pg/ml, *t* = 6.979, *P* = 0.000; IL-6: 248.01 ± 5.81 pg/ml vs. 154.41 ± 8.39 pg/ml, *t* = 9.167, *P* = 0.000; TNF-α: 514.41 ± 23.90 pg/ml vs. 321.78 ± 10.09 pg/ml, *t* = 7.423, *P* = 0.000) **p < 0.001. IL, interleukin; TNF, tumor necrosis factor.

**Figure 5:**
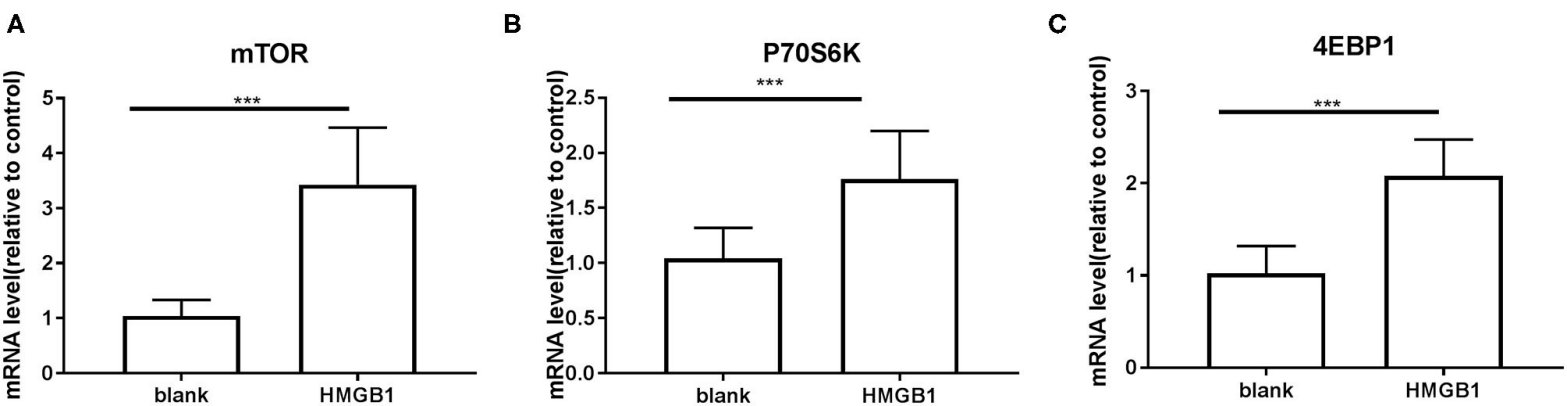
mRNA expression of mTOR and its substrates P70S6K and 4EBP1 on mDCs measured by RT-PCR. Application of the 2^−Δ*ΔCT*^ method. The expression of mTOR **(A)**, P70S6K **(B)**, and 4EBP1 **(C)** were up-regulated in mDCs after stimulation with HMGB1. (mTOR: 1.039 ± 0.0651 vs. 3.428 ± 0.2315, *t* = 9.935, *P* = 0.000; P70S6K: 1.042 ± 0.06223 vs. 1.763 ± 0.09726, *t* = 6.245, *P* = 0.000 4EBP1: 1.021 ± 0.066 vs. 2.078 ± 0.08861, *t* = 9.546, *P* < 0.000.) ***P < 0.001.

The mDCs from 20 SLE patients were isolated and induced to mature with cytokines, then stimulated with HMGB1. Compared to the healthy control group, mDCs stimulated by HMGB1 expressed more HLA-DR, CD40, and CD86 in SLE patients (Figure 3) and produced significantly more TNF-α, IL-6, and IL-1β (Figure 4). Moreover, HMGB1 induced up-regulation of mTOR and its substrates (P70S6K, 4EBP1) in mDCs (Figure 5). Therefore, HMGB1 can induce activation of mDCs from patients with SLE and up-regulate mTOR pathway in mDCs.

### Activation of mDCs Could Be Inhibited by Blocking the mTOR Pathway With Rapamycin

**Figure 6:**
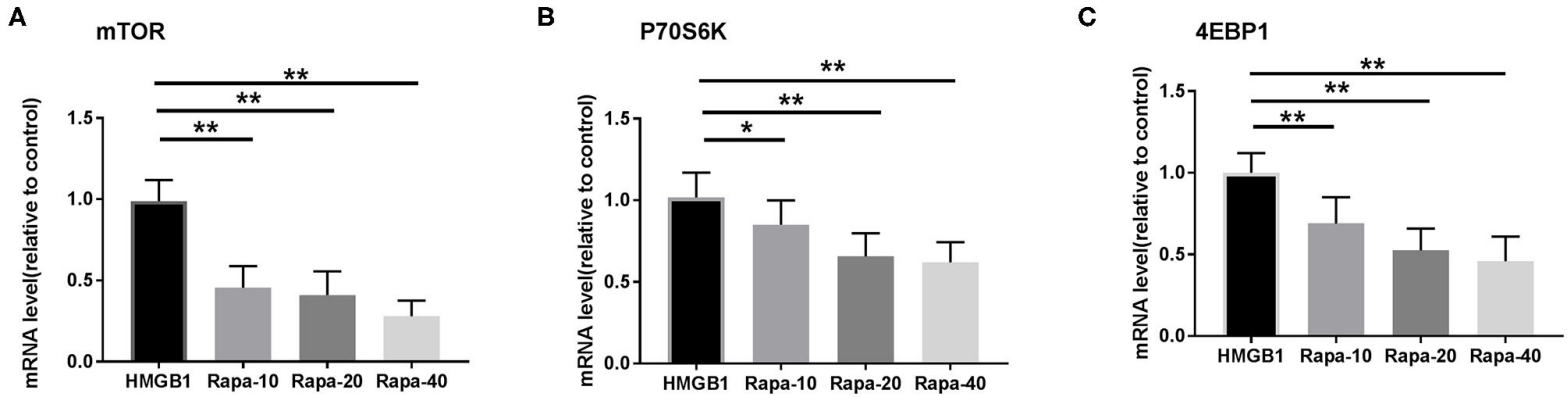
mRNA expression of mTOR and its substrates P70S6K and 4EBP1 in mDCs under different concentrations of rapamycin (Rapa-10: 10 ng/ml; Rapa-20: 20 ng/ml; Rapa-40: 40 ng/ml) intervention by RT-PCR. Application of the 2^−Δ*ΔCT*^ method. The expression of mTOR **(A)**, P70S6K **(B)**, and 4EBP1 **(C)** were significantly decreased in mDCs through Rapa intervention. *p < 0.05, **p < 0.01.

**Figure 7:**
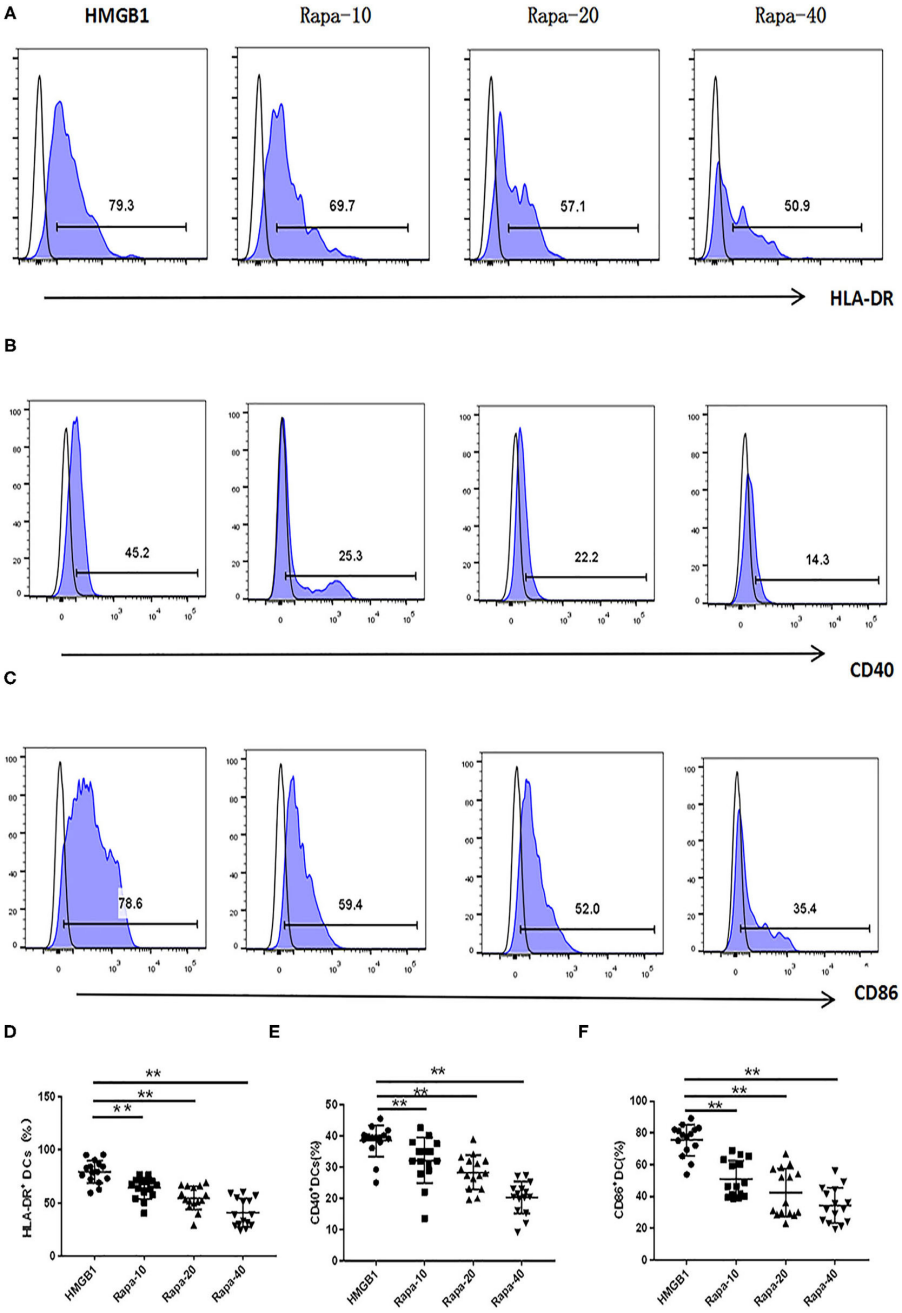
Rapamycin can inhibit HMGB1-induced mDC expression of HLA-DR, CD40 and CD86 increase. Different concentrations of rapamycin (Rapa-10: 10 ng/ml; Rapa-20: 20 ng/ml; Rapa-40: 40 ng/ml) were put into cultures of primary mDCs. Flow cytometry analysis of HLA-DR **(A)**, CD40 **(B)** and CD86 **(C)**, surface expression on each group mDCs from SLE patients. The expression of HLA-DR **(D)**, CD40 **(E)**, and CD86 **(F)** were decreased on mDCs through Rapa intervention compared with HMGB1 group. **P < 0.01.

**Figure 8:**
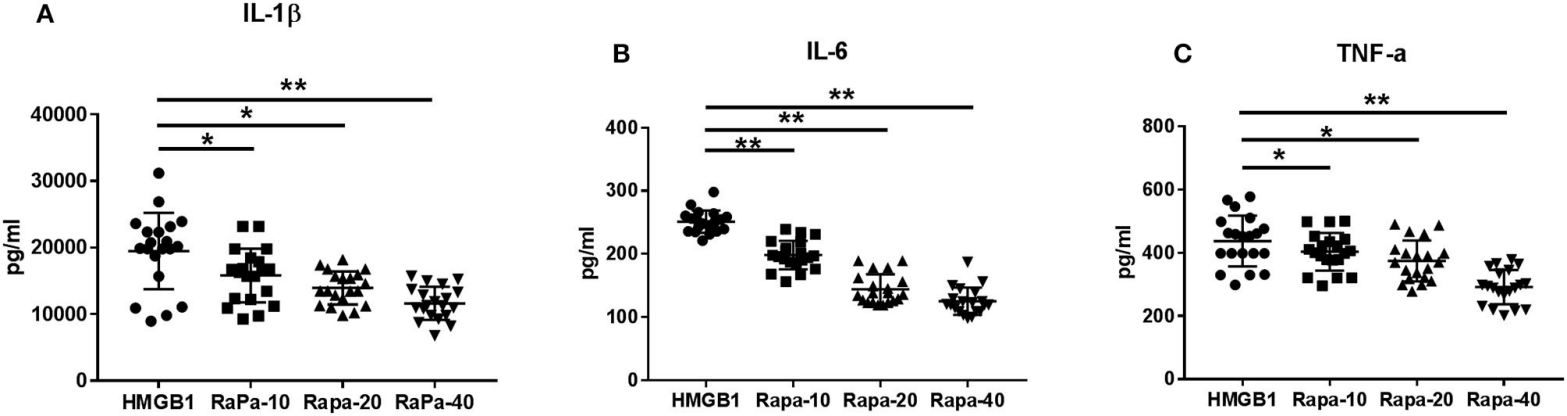
Cytokine production through Rapa intervention. mDCs from systemic lupus erythematosus (SLE) patients were blocked by different concentrations of rapamycin, and production of IL-1β **(A)**, IL-6 **(B)**, and TNF-α **(C)** were measured by ELISA. Different concentrations of rapamycin (Rapa-10: 10 ng/ml; Rapa-20: 20 ng/ml; Rapa-40: 40 ng/ml) were added into cultures of primary mDCs. Data are presented as the median and interrogative range. *p < 0.05, **p < 0.01. IL, interleukin; TNF, tumor necrosis factor.

We inhibited the mTOR pathway with different concentrations of rapamycin (Rapa). The mRNA expression of mTOR and its substrate P70S6K and 4EBP1 in mDCs was considerably decreased through Rapa intervention by RT-PCR (Figure 6 and Supplementary Table 2). These results suggested that rapamycin can inhibit the activation of the mTOR pathway in mDCs induced by HMGB1. Furthermore, rapamycin can inhibit increased expression of HLA-DR, CD40, CD86 (Figure 7 and Supplementary Table 3) and increased secretion of TNF-α, IL-6, and IL-1 induced by HMGB1 (Figure 8 and Supplementary Table 4), respectively. Overall, these results suggested that HMGB1-induced activation of mDCs could be affected by blocking the mTOR pathway with rapamycin.

## Discussion

SLE, a systemic autoimmune disease, is a potentially fatal disease characterized by immune complex deposition and the subsequent inflammation that contribute to severe tissue damage (21). Recent reports show that HMGB1 might be involved in autoimmune and inflammatory diseases, including SLE (22–24). Previous studies and our results revealed that the level of HMGB1 was positively correlated with SLEDAI score in SLE patients, implying that the critical role of HMGB1 in the pathogenesis of SLE.

Recent studies indicated that HMGB1, a well-established DAMP, is responsible for triggering inflammatory responses (7). HMGB1 is likely to be released from activated immune cells such as macrophages in the area of inflammation or injury (12, 13). In this study, we found that mDCs were activated in SLE. The levels of CD40 and CD86 were positively correlated with SLEDAI scores, and the activation of mDCs was associated with up-regulation of HMGB1 and mTOR. The expression of mTOR in mDCs increased in SLE, and the levels of mTOR in mDCs were positively correlated with SLEDAI scores and the levels of HMGB1 in peripheral blood, respectively. These findings implied that HMGB1 might induce activation of mDCs in patients with SLE and up-regulation of the mTOR pathway.

In this study, the results indicated that HMGB1 can activate dendritic cells and induce more proinflammatory cytokines (TNF-α, IL-6, and IL-1β). These results were consistent with previous studies (25). Persistent elevation of proinflammatory cytokines could lead to immune deregulation followed by local inflammatory processes and tissue damage (26, 27). In another study (28), the surface level of CD86 on monocytes in SLE was comparable with that in HCs. CD163 is an anti-inflammatory marker, whereas HLA-DR is a proinflammatory marker. These data demonstrated the downregulation of proinflammatory surface markers but the upregulation of anti-inflammatory markers in SLE, which was different from the presumptive results. This discrepancy might be due to an existing negative feedback to maintain monocyte homeostasis in SLE.

mTOR plays a crucial role in the relationship among HMGB1, activation of mDCs, and autoimmunity in SLE. In this study, when stimulated by HMGB1, mDCs expressed increased mTOR and its substrates (P70S6K, 4EBP1) compared to the healthy control group. When the mTOR pathway was blocked with different concentrations of rapamycin (Rapa), HMGB1-induced mDC expression of HLA-DR, CD40, and CD86, and proinflammatory cytokines secretion was decreased. All these results implied that HMGB1 may induce activation of mDCs through the up-regulated mTOR pathway, but further research is needed to confirm this hypothesis.

It is well-known that mTOR signaling senses extracellular stimulations and regulates many biological processes including inflammation (29). Activation of mTOR delivers an obligatory signal for the proper activation and differentiation of mDCs in our present study. Therefore, the activation of mTOR signaling pathway is a potentially significant factor contributing to the pathogenesis of SLE. A recent study found that mTOR signaling activation caused hyperpolarization of mitochondria, resulting in necrosis tendency of T cells, promoting the generation of anti-nuclear antibodies, activation of dendritic cells, and the occurrence of inflammation (30).

In the present study, we intended to study upstream regulators of the mTOR pathway and its function in mDCs. Our data showed that rapamycin can inhibit HMGB1-induced activation of mDCs and secretion of pro-inflammatory cytokines. The next question would be to clarify the pathophysiological functions of mTOR in SLE and investigate targeted drugs. Given the general importance of the mTOR signaling pathway and considering the ubiquitous expression of HMGB1, the newly uncovered regulation is expected to have a broad impact. It includes an impact on metabolic programs and cell fate decisions in other immune and non-immune cells under homeostasis, when faced with an environmental challenge, and during the development of the autoimmune disease.

Therefore, blocking mTOR signaling pathway becomes a new target for the treatment of SLE. Rapamycin, a widely recognized blocker of the mTOR pathway, has a promising prospect in treating SLE. Rapamycin can not only inhibit the signal transduction downstream of mTOR, but also negatively regulate the PI3K/AKT /mTOR pathway. The monotherapy of rapamycin can completely prevent nephritis in mice and significantly improve the condition. Gu's study (31) demonstrated that RAPA alleviated the clinical symptoms of lupus nephritis and prolonged survival in MRL/lpr mice. This result is consistent with our previous research (32).

There are some limitations to our research. First, all the patients in the study had moderate severity of SLE. Thus, the findings may not be generalizable to patients with mild SLE. Second, we used HMGB1 to stimulate myeloid-derived dendritic cells (mDCs). However, mature mDCs cannot be separated directly from peripheral blood. Instead, PBMCs were isolated and induced to mature mDCs with the cytokine. The mDCs in this study shared some similar characteristics with those in peripheral blood. The degree of similarity between these mDCs cultured *in vitro* and those *in vivo* warrants further investigation. Lastly, the effect of HMGB1- activated dendritic cells on T cells was not evaluated, which is needed further investigation. However, HLA-DR and costimulatory molecules of dendritic cells and cytokine synthesis were measured to estimate the activation of dendritic cells.

In summary, our study found that the mDCs were activated in the peripheral blood of SLE. HMGB1 induces dendritic cell activation and up-regulate the mTOR pathway in SLE. Rapamycin can inhibit HMGB1-induced activation of mDCs and secretion of pro-inflammatory cytokines. These findings indicate that targeting mTOR pathway could be a novel therapeutic approach to SLE.

## Data Availability Statement

The original contributions presented in the study are included in the article/Supplementary Material, further inquiries can be directed to the corresponding author.

## Ethics Statement

The studies involving human participants were reviewed and approved by Medical Ethical Committee of the First Affiliated Hospital of Guangxi Medical University. The patients/participants provided their written informed consent to participate in this study.

## Author Contributions

XS, HZ, YZ, YL, and QT conducted the study and collected the data. XS, XiaZ, and HZ were involved in study design, data analyses, and writing and editing of the manuscript. All authors contributed to the article and approved the submitted version.

## Conflict of Interest

The authors declare that the research was conducted in the absence of any commercial or financial relationships that could be construed as a potential conflict of interest.

## Supplementary Material

Supplementary Figure 1 CD11c + DC purity tests before and after cell culture. (**A**: CD11c + DC purity tests before cell culture; **B**: CD11c + DC purity tests before cell culture).

Supplementary Table 1 Characteristics of systemic lupus erythematosus (SLE) patients and healthy control subjects.

Supplementary Table 2 mTOR and its substrate P70S6K and 4 ebp1 mRNA expression on dendritic cells through Rapa intervention.

Supplementary Table 3 HLA-DR, CD40, and CD86 in dendritic cells supernatant through Rapa intervention (%).

Supplementary Table 4 Cytokines in dendritic cells supernatant through Rapa intervention (pg/ml).

## Abbreviations

DCs: dendritic cells
SLE: Systemic lupus erythematosus
mDCs: myeloid dendritic cells
HMGB1: high-mobility group box protein-1
mTOR: mechanistic target of rapamycin
HLA-DR: human leukocyte antigen DR
DAMP: damage associated molecular pattern
RAGE: receptor of advanced glycation end products
ACR: American College of Rheumatology
PBMC: Peripheral blood mononuclear cells
Rapa: rapamycin
HCs: healthy controls.
