# Supplementary figures and images for: HMGB1 Activates Myeloid Dendritic Cells by Up-Regulating mTOR Pathway in Systemic Lupus Erythematosus
Source: Front Med (Lausanne). 2021 Jun 7;8:636188. doi: 10.3389/fmed.2021.636188 (PMC8215142; doi:10.3389/fmed.2021.636188)

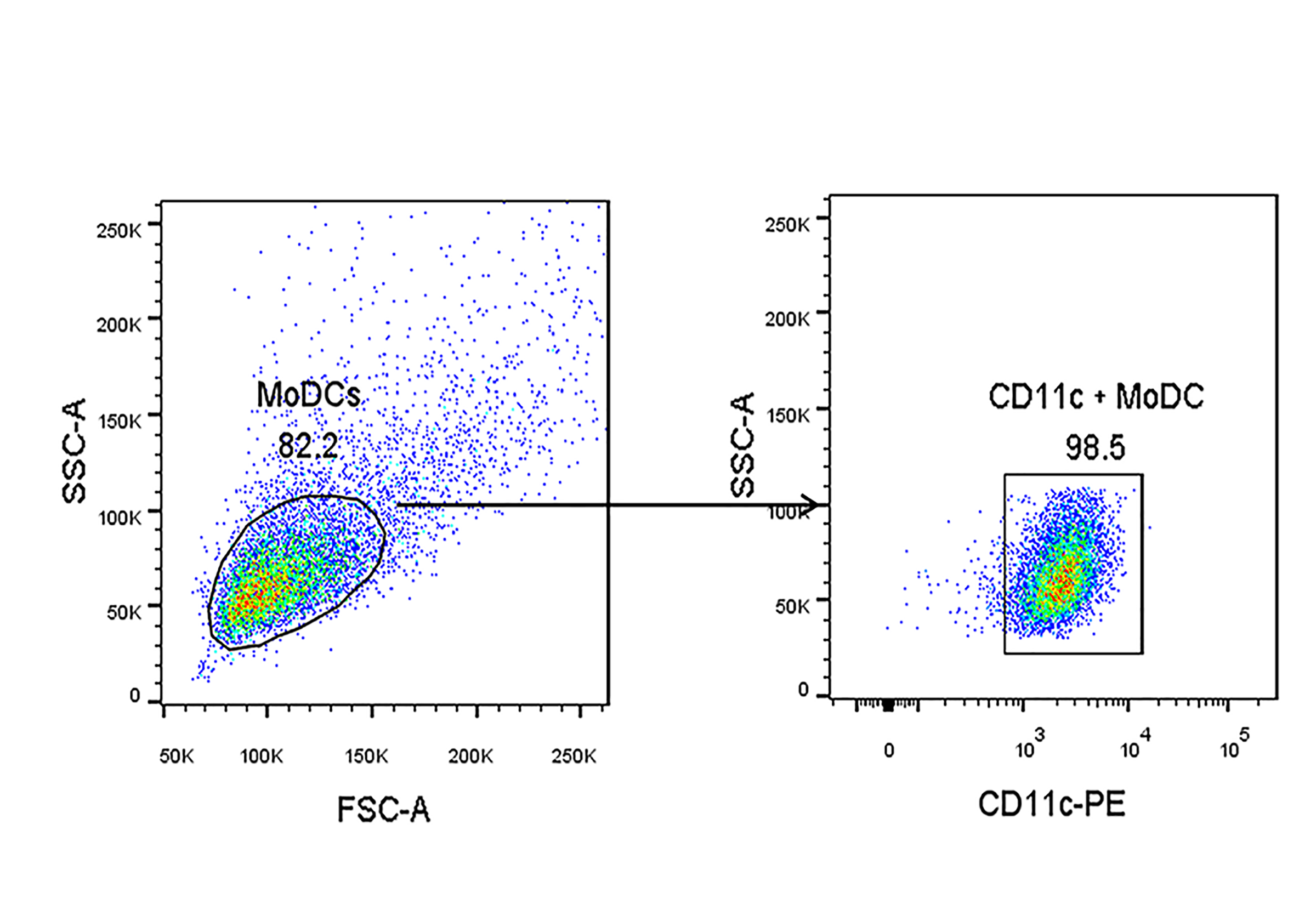

Supplement: Supplementary Figure 1 — CD11c + DC purity tests before and after cell culture. (A: CD11c + DC purity tests before cell culture; B: CD11c + DC purity tests before cell culture). [file Image_1.TIF]
