# Supplementary material for: HMGB1 Activates Myeloid Dendritic Cells by Up-Regulating mTOR Pathway in Systemic Lupus Erythematosus
Source: Front Med (Lausanne). 2021 Jun 7;8:636188. doi: 10.3389/fmed.2021.636188 (PMC8215142; doi:10.3389/fmed.2021.636188)
Supplement: Supplementary Table 1 — Characteristics of systemic lupus erythematosus (SLE) patients and healthy control subjects. [file Table_1.DOCX]

the online supplementary table 1. Characteristics of systemic lupus erythematosus (SLE) patients and healthy control subjects.

|  | SLE  (n = 35) | HCs  (n = 20) |
| --- | --- | --- |
| Female (n, %) | 29 (83%) | 12 (60%) |
| age(year, mean ± s.d.) | 40.00±13.27 | 38.16±12.46 |
| Anti-dsDNA (Positive/Negative ) | 9 (26%) | n.a. |
| Anti-sm (Positive/Negative ) | 15 (43%) | n.a. |
| Rush (n, %) | 17 (49%) | n.a. |
| injury of hematologic system (n, %) | 14 (40%) | n.a. |
| Patients with nephritis (n, %) | 23 (66%) | n.a. |
| interstitial pneumonia(n, %) | 5(14%) | n.a. |
| pulmonary artery hypertension | 2(6%) | n.a. |
| hsCRP(mg/L, mean ± s.d.) | 22.94±39.79 | n.a. |
| Complement C3 | 0.68±0.29 | n.a. |
| Complement C4 | 0.19±0.26 | n.a. |
| SLEDAI score (mean ± s.d.) | 9.9±4.0 | n.a. |
| Corticosteroids treatment | none | none |
| Immunosuppressive treatment | none | none |

Values are in mean±standard deviation (s.d.); n.a.: not applicable.

**Table 2 mTOR and its substrate P70S6K and 4 ebp1 mRNA expression on dendritic cells** **through Rapa intervention**

| GENE | GROUP | N | 2^-ΔΔCT^ | T^*^ | P^*^ |
| --- | --- | --- | --- | --- | --- |
| mTOR | HMGB1 | 20 | 0.988±0.130 | - | - |
|  | Rapa-10 | 20 | 0.455±0.132 | 15.263 | 0.000 |
|  | Rapa-20 | 20 | 0.410±0.146 | 13.678 | 0.000 |
|  | Rapa-40 | 20 | 0.280±0.096 | 10.661 | 0.000 |
| P70S6K | HMGB1 | 20 | 1.017±0.152 | - | - |
|  | Rapa-10 | 20 | 0.851±0.149 | 34.587 | 0.000 |
|  | Rapa-20 | 20 | 0.657±0.141 | 22.744 | 0.000 |
|  | Rapa-40 | 20 | 0.620±0.123 | 21.272 | 0.000 |
| 4EBP1 | HMGB1 | 20 | 1.000±0.120 | - | - |
|  | Rapa-10 | 20 | 0.689±0.162 | 25.323 | 0.000 |
|  | Rapa-20 | 20 | 0.525±0.133 | 17.763 | 0.000 |
|  | Rapa-40 | 20 | 0.459±0.151 | 15.106 | 0.000 |

* T value and P value were the comparison of mRNA expression in HMGB1 group with different concentrations of RAPA group.

**Table 3 HLA-DR,CD40 and CD86 in dendritic cells supernatant through Rapa intervention(%）**

| phenotype | GROUP | N | positive rate（x±s）% | T* | P* |
| --- | --- | --- | --- | --- | --- |
| HLA-DR | HMGB1 | 15 | 79.27±10.78 | - | - |
|  | Rapa-10 | 15 | 63.84±10.00 | 30.420 | 0.000 |
|  | Rapa-20 | 15 | 54.63±11.10 | 22.207 | 0.000 |
|  | Rapa-40 | 15 | 40.80±13.36 | 14.351 | 0.000 |
| CD40 | HMGB1 | 15 | 38.42±5.10 | - | - |
|  | Rapa-10 | 15 | 32.23±7.27 | 27.933 | 0.000 |
|  | Rapa-20 | 15 | 28.40±5.44 | 25.182 | 0.000 |
|  | Rapa-40 | 15 | 20.25±5.17 | 15.265 | 0.000 |
| CD86 | HMGB1 | 15 | 77.7±4.6 | - | - |
|  | Rapa-10 | 15 | 42.6±3.7 | 18.908 | 0.000 |
|  | Rapa-20 | 15 | 30.9±4.6 | 22.846 | 0.000 |
|  | Rapa-40 | 15 | 28.5±5.1 | 22.830 | 0.000 |

* T value and P value were the comparison of mRNA expression in HMGB1 group with different concentrations of RAPA group.

**Table 4 cytokines in dendritic cells supernatant through Rapa intervention(pg/ml)**

| cytokines | GROUP | N | concentration（x±s） | T* | P* |
| --- | --- | --- | --- | --- | --- |
| IL-1β | HMGB1 | 20 | 19491±5736.76 | - | - |
|  | Rapa-10 | 20 | 15814±4045.62 | 21.301 | 0.000 |
|  | Rapa-20 | 20 | 13935±2471.68 | 20.371 | 0.000 |
|  | Rapa-40 | 20 | 11631±2468.80 | 16.674 | 0.000 |
| IL-6 | HMGB1 | 20 | 251.11±17.57 | - | - |
|  | Rapa-10 | 20 | 144.23±22.55 | 42.628 | 0.000 |
|  | Rapa-20 | 20 | 136.05±23.54 | 21.602 | 0.000 |
|  | Rapa-40 | 20 | 125.15±21.63 | 17.844 | 0.000 |
| TNF-α | HMGB1 | 20 | 437.13±80.34 | - | - |
|  | Rapa-10 | 20 | 403.49±59.58 | 36.991 | 0.000 |
|  | Rapa-20 | 20 | 374.32±64.38 | 32.652 | 0.000 |
|  | Rapa-40 | 20 | 291.37±54.49 | 22.993 | 0.000 |

* T value and P value were the comparison of mRNA expression in HMGB1 group with different concentrations of RAPA group.
